# Cre/LoxP Genetic Recombination Sustains Cartilage Anabolic Factor Expression in Hyaluronan Encapsulated MSCs Alleviates Intervertebral Disc Degeneration

**DOI:** 10.3390/biomedicines10030555

**Published:** 2022-02-26

**Authors:** Long-Yi Chan, Cheng-Chung Chang, Po-Liang Lai, Tomoji Maeda, Horng-Chaung Hsu, Chin-Yu Lin, Shu-Jui Kuo

**Affiliations:** 1 Institute of New Drug Development, College of Medicine, China Medical University, Taichung 40402, Taiwan; longlong1993429.lyc@gmail.com (L.-Y.C.); johnchang.hb@gmail.com (C.-C.C.); 2 Department of Orthopedic Surgery, Chang Gung Memorial Hospital, Tau-Yuan 333, Taiwan; polianglai@gmail.com; 3 Tsuzuki Institute for Traditional Medicine, College of Pharmacy, China Medical University, Taichung 40402, Taiwan; t-maeda@nichiyaku.ac.jp; 4 Department of Pharmaceutical Sciences, Nihon Pharmaceutical University, Kitaadachi-gun, Saitama 362-0806, Japan; 5 School of Medicine, China Medical University, Taichung 40402, Taiwan; d4749@mail.cmuh.org.tw; 6 Department of Orthopedic Surgery, China Medical University Hospital, Taichung 40447, Taiwan; 7 Master Program for Biomedical Engineering, Collage of Biomedical Engineering, China Medical University, Taichung 40402, Taiwan

**Keywords:** runx1 transcription factor, baculoviral vector, gene therapy, Cre/LoxP gene editing, disc degeneration

## Abstract

(1) Background: Inexplicable low back and neck pain frequently results from spinal disc degeneration with an imbalanced intervertebral disc (IVD) cell homeostasis. We hypothesize that introducing MSC expressing a sustained cartilage-anabolic factor in the IVD may stimulate the mucoid materials secreted from the IVD cells, promote the MSC’s chondrogenesis and maintain the hydration content providing mechanical strength to decelerate the disc degeneration progression; (2) Methods: This study expressed a cartilage-anabolic factor runx1 by a baculoviral vector (BV) transduced MSCs through a Cre/LoxP gene editing and recombination system for sustained recombinant runx1 transcription factor production. The Cre/LoxP BV modified MSCs were encapsulated by hyaluronan hydrogel, due to its’ vital composition in ECM of a healthy disc and transplanted to a punctured coccygeal disc in rats through micro-injection, followed by X-ray radiography and histological analysis at the 4- and 12-weeks post-transplantation; (3) Results: Data reveals the Cre/LoxP BV system-mediated long-termed runx1 gene expression, possessing good biosafety characteristics in the in vitro cell transduction and in vivo MSCs transplantation, and maintained superior hydration content in the disc than that of mock transduced MSCs; (4) Conclusions: This proof-of-concept study fulfills the need of implanting therapeutic cells accompanied with microinjection in the disc, such as a discography and paves a road to manufacture composite hyaluronan, such as peptide modified hyaluronan as an MSC carrier for IVD regeneration in the future study.

## 1. Introduction

With global aging population, spine pathologies remain one of the leading causes of disability and financial burden, although the medical devices development and healthy technology were largely improved in recent years. Low back pain was one of the top three leading causes worldwide contributors of Years Lived with Disability (YLD) from 1990 to 2017 [1], affecting approximately ~632 million people globally. The economic burden of low back and neck problems in the USA is $85.9 billion, higher than that of many other causes of disability, including arthritis, and potentially fatal diseases such as cancer [2,3]. Inexplicable lumbar back or neck pain is frequently associated with intervertebral disc (IVD) damage, and imaging studies have revealed the relationship between prevalent IVD injury elicited degeneration and subsequent severity of body back pain [4,5]. IVDs are the most crucial part of the healthy spinal column by providing stability while permitting motion between the vertebrae [6]. Notably, the complex structural features of IVDs enable them to absorb and disperse physical weight-loading from motion activities and other body parts. The IVD is composed of a central hydrophilic proteoglycan-rich gelatinous matrix, the nucleus pulposus (NP), which is surrounded by a multilamellar collagenous ring, the annulus fibrosus (AF), and cartilaginous bony end-plates that separate the discs from the vertebrates [7]. The tight AF ring protects the high osmotic pressure within the NP, enabling the IVDs to absorb compressive forces [8]. The gelatinous matrix-specific NP cells provide the extracellular matrix (ECM) components of the IVDs and maintain homeostasis.

Runx1 is directly involved in kartogenin-mediated cartilage regeneration and protection and induces chondrogenesis through regulating the nuclear localization of the core-binding factor-beta (Cbfβ) transcription complex [9]. Moreover, runx1 has also been demonstrated to control the type II collagen expression by directly binding with its promoter responsive elements [10] and has been shown to play a critical role in the control of MSC chondrogenesis and chondrocyte proliferation and survival [11,12]. Previously, we have created a rat disc puncture model, followed by administrating runx1 expressing mRNA alone. Our data demonstrated that the runx1 recombinant protein produced in the disc puncture area ameliorates the progress of disc degeneration [13]. However, the damaged disc could not acquire satisfactory healing efficacy, which might attribute to a shortage of runx1 expression and lack of multipotent cells supplementation.

Furthermore, gene therapy combined with MSC delivery has been successfully applied on regenerative medicine, superior to that of directly administrating therapeutic protein in the damaged tissue and has been extensively investigated in both preclinical and clinical studies. Baculoviruses are a diverse group of enveloped DNA viruses capable of infecting more than 700 insect species and are prevalently utilized for recombinant protein production [14]. Yet, it can also effectively transduce many mammalian cells, the so-called Bac-Mam gene delivery system [15,16,17]. Baculovirus transduction neither causes appreciable cytotoxicity nor replicates within mammalian cells; non-pathogenic recombinant baculovirus vector is easily constructed and largely scaled-up produced in biosafety level 1 facilities [18]. The high safety characteristic and gene transduction efficiency of the Bac-Mam system attributed it emerge as one of the popular gene delivery vehicles for mammalian cells, combined with tissue engineering for regenerative medicine. However, the shortage of recombinant protein expression from baculoviral gene transduction in mammalian cells makes it challenging to apply to chronic diseases, such as IVDs homeostasis reconstruction. In our previous study, we modified the baculoviral vector (BV) and utilized the FLP/Frt gene recombinant mechanism to elongate the duration of the transgene expression [19]. To improve the efficiency of transgene targeting and recombination, we introduced the Cre/LoxP site-specific recognition machinery into a baculoviral vector, attempting to achieve a more sustained transgene expression duration [20]. This higher mammalian cells transduction rate is advantageous for non-secreted proteins, such as the intracellular transcription factor runx1. Since runx1 recombinant protein was approved to ameliorate the disc degeneration scenario, whether combined with genetically modified MSCs using Cre/LoxP gene-editing system can sustain the runx1 expression and facilitate the seriously damaged disc healing rate is yet to be explored.

In the current study, we hypothesized that allotransplantation of genetically modified MSCs sustained overexpressing runx1 recombinant protein in the traumatized disc may hold dual functions, which not only secretes abundant cartilage-anabolic factor, stimulates mucoid material produced from resident NP tissue, but also keeps the differentiation capability of MSCs. Overall maintain the hydration content to provide mechanical strength and re-supplement multi-potent stem cells to offer regeneration capability to improve the disc healing progress. Here we proved this hypothesis in a traumatic injury-induced disc degeneration model in rats and utilized dual recombinant Cre/LoxP baculoviruses transduction for sustained expression of runx1 in MSCs. Both sustained and conventional runx1 expressed MSCs were sophisticatedly encapsulated in hyaluronan and separately microinjected to the punctured coccygeal disc, compared with control animals that received mock-transduced MSCs. Disc degeneration progress was evaluated by X-ray radiography to measure disc height and immunohistological examination to monitor the exogeneous runx1 expression, extracellular matrix composition, and consequent inflammatory responses. Meanwhile, sustained gene expression scenarios, cytotoxicity, pro-inflammatory cytokines expression, and chondrogenic differentiation mediated by Cre/Loxp assisted gene overexpression were also evaluated. This study provides a proof-of-concept study to examine disc degeneration treatment by hyaluronan delivery of gene-edited MSCs for overexpressing cartilage anabolic factors and conveys an approach to connect primary findings for clinical disease modification.

## 2. Materials and Methods

### 2.1. Construction of Recombinant Baculovirus and Titer Determination

All transgenes were expressed under the control of cytomegalovirus immediate-early (CMV-IE) promoter. Bac-Cre expressed Cre recombinase was generously acquired from Prof. Hu, constructed previously [20]. Bac-Luc2 and Bac-L-Luc2 expressed short-termed and long-termed photinus pyralis luciferase were subcloned from pGL4 plasmid (Promega, Madison, WI, USA. Cat. E6651) through retrieving the *luc2* cDNA with optimized restriction sites to generate the corresponding pFastBac donor plasmids, followed by Bac-to-Bac^®^ manufacturing process to produce the recombinant BVs [20]. Bac-Runx1 and Bac-L-Runx1 expressed short-termed and long-termed runx1 transcription factors were sub-cloned from the pSP73-Runx1-FL vector [21], followed by an identical process to produce the recombinant BVs. BV-EGFP expressed enhanced green fluorescent protein (EGFP) was subcloned from the pAcGFP1 vector (Cat. 632468, Clontech, Mountain View, CA, USA). Hybrid BV vectors Bac-L-Luc2 and Bac-L-Runx1 accommodated the LoxP-flanking *luc2* and *runx1* genes, respectively. All recombinant baculoviruses were amplified and tittered by the end-point dilution method [22].

### 2.2. Cell Culture, Baculoviral Transduction, Western Blot, and Luminescence Examination

All animal experiments were approved by the China Medical University Institutional Animal Care and Use Committee (IACUC), with approval number: CMUIACUC-2019-159, and were performed in compliance with the Guide for the Care and Use of Laboratory Animals (National Laboratory Animal Center, NARLabs, Taipei, Taiwan). The bone marrows derived MSCs (BMSCs) were collectively mixed and harvested from the femora and tibias of 2–3 SD rats using a standard protocol as previously described [23,24,25,26], which also has been demonstrated the integrity and correctness of the CD markers in the isolated MSCs [27]. Briefly, the bone marrow was flushed entirely out from the femora and tibias with Hanks’ Balanced Salt Solution (HBSS) (Cat. 14025092, ThermoFisher Scientific, Allentown, PA, USA), collected in 50 mL tube, followed by several times pipetting to remove the blood clots. The cells were separated using Histopaque^®^-1077 (Cat. 10771, Sigma-Aldrich, St. Louis, MO, USA) after centrifugation (900× *g*, 30 min). The nucleated cells were collected from the Histopaque^®^/plasma interface, diluted with two volumes of HBSS, centrifuged, and resuspended in Dulbecco’s modified Eagle medium (DMEM, Invitrogen, Carlsbad, CA, USA) containing 10% fetal bovine serum (FBS, Thermo Scientific Hyclone, Rockford, IL, USA), 100 IU/mL penicillin and 100 IU/mL streptomycin. The cells were plated into tissue culture flasks (≈2 × 10^5^ cells/cm^2^) and incubated in 5% CO_2_ at 37 °C. After 4–5 days, non-adherent cells were removed, and the adherent cells continued to be cultured for 2–4 days until the formation of colonies. Then, the cells (BMSCs) were resuspended and replated into a new flask for subculture until 90% confluence and termed as P1. The BMSCs were passaged, and cells of passage 3 or 4 were used.

To examine the BMSCs differentiation, BMSCs cultured in 6-well plates were supplemented with osteogenic medium (Cat. A1007201, ThermoFisher Scientific, USA) and chondrogenic medium (Cat. A1007101, ThermoFisher Scientific, USA), respectively. Operation in compliance with manufacturer’s instruction, followed by Alizarin Red staining (Cat. TMS-008-C, Sigma-Aldrich, USA) and Alcian blue staining (Cat. TMS-010, Sigma-Aldrich, USA). The cells were observed, and images were captured under stereo microscopy. For co-transduction with Bac-Cre/Bac-L-Luc2 or Bac-Cre/Bac-L-Runx1, BMSCs cultured in 6-well plates or T-75 flasks were incubated with Bac-Cre (MOI 100) and the hybrid vector (Bac-L-Luc2 or Bac-L-Runx1, MOI 100 for 2 h using NaHCO_3_-free DMEM as the surrounding solution such as the transduction protocol described previously [28]. Following transduction, the cells were cultured in a complete medium containing 3 mM sodium butyrate (Cat. 303410, Sigma-Aldrich, USA). After 12 h incubation, the medium was replaced by a standard complete medium and was exchanged completely every 3 days for subsequent experiments. 

To analyze runx1 expressed from recombinant BV transduction, BMSCs were cultured in 4 × 10^5^ cells in 6-well overnight, and transduced in MOI 100 in compliance with the protocol described above. Cell’s protein was extracted with RIPA lysis buffer (Cat. 20-188, Merck millipore, Burlington, MA, USA) containing 0.2% phosphatase inhibitor cocktail (Cat. P2850, Sigma-Aldrich, USA), phenylmethylsulfonyl fluoride (Cat. 52332, Sigma-Aldrich, USA) at 4 °C for 1 h, 15,000 rpm centrifugation 15 min for protein quantification using Pierce^TM^ BCA Protein Assay Kit (Cat. 23225, ThermoFisher Scientific, USA). Proteins (10 μg) were electrophoresed by SDS-polyacrylamide gel and transferred to a PVDF (poly (vinylidene fluoride)) (Cat. BSP0861, Pall Corporation, Port Washington, NY, USA) membrane. The membrane was blocked in 5% fat-free milk in PBST (PBS with 0.05% Tween-20), followed by incubation overnight with the following primary antibodies diluted in PBST: Runx1 (D-2) (Cat. sc-365644, Santa Cruz, Dallas, TX, USA) and GAPDH (Cat. sc-32333, Santa Cruz, CA, USA) (diluted to 1:1000). The primary antibodies were removed, and the membrane was washed extensively in PBST. Subsequent incubation with horseradish peroxidase-conjugated goat anti-mouse antibodies (1:20,000, Cat. sc-2005, Santa Cruz, CA, USA) was performed at room temperature for 2 h. The membrane was washed extensively in PBST to remove excess secondary antibodies, and the blot was visualized with enhanced chemiluminescence reagent (Cat. GERPN2209, GE Healthcare, Chicago, IL, USA).

To examine the transduction efficiency of recombinant BV, BMSCs were seeded in 4 × 10^5^ cells in 6-well overnight, transduced with BV-EGFP in MOI 100 using NaHCO_3_-free DMEM as surrounding medium. At 1 day post-transduction, BMSCs were trypsinized and subjected to a flow cytometer (FACSCanto II, BD Biosciences, Franklin Lakes, NJ, USA), the green fluorescence intensity and percentage were examined. To measure in vitro firefly Luc2 expression, cell lysate was collected at 2–13 days post-transduction, bioluminescence was measured on SpectraMax iD3 Multi-mode Microplate Reader (Molecular Devices, San Jose, CA, USA) using the Pierce™ Firefly Luciferase Assay kit (Cat.16174, ThermoFisher Scientific, USA) in compliance with manufacturer’s instructions.

### 2.3. Analysis of Cell Viability, Proliferation, and qRT-PCR

For cell viability and proliferation assay, BV transduced BMSCs in 4 × 10^4^ cells in 96 well plates were collected at time points as indicated in results, cell mediums were examined by MTS-1 Cell Proliferation Colorimetric Assay Kit (Biovision, Cat. K300) and detected absorbance at O.D. 450 nm in compliance with manufacturer’s instructions. For qRT-PCR gene expression measurement, total RNA from BMSCs post-transduction was extracted by RNeasy mini kit (Cat. 74106, QIAGEN, Germantown, MD, USA) in compliance with the manufacturer’s instruction. cDNA was synthesized by High-Capacity cDNA Reverse Transcription Kit (Cat. 4368814, Applied Biosystems, San Francisco, CA, USA), and qRT-PCR was performed by Fast SYBR™ Green Master Mix (Cat. 4385616, Applied Biosystems, USA) through QuantStudio™ 3 Real-Time PCR System (Cat. A28567, ThermoFisher Scientific, USA). The primer pairs used in qRT-PCR as following: IL-1β_F cagaattgccattgcacaac, IL-1β_R aaagaaggtgcttgggtcct, IL-6_F ccggagaggagacttcacag, IL-6_R cagaattgccattgcacaac, TNF-α_F cgtgcagccagttgtctaaa, TNF-α_R atctccctcgtctcccatct, GAPDH_F tgccactcagaagactgtgg, GAPDH_R ttcagctctgggatgacctt, COL2A1_F cgaggtgacaaaggagaagc, COL2A1_R agggccagaagtaccctgat, COL10A1_F agccaggctatggaagtcct, COL10A1_R agctgggccaatatctcctt, Cbfβ_F caaacacctagccgggaata, Cbfβ_R gcaacccataccatccaatc, Sox9_F ctgaagggctacgactggac, Sox9_R tactggtctgccagcttcct, Runx1_F ctcagcggaactttccagtc, Runx1_R caggaggcgagtaggtgaag. The primer pairs used in semi-quantitative PCR are as follows: BV/Runx1-mini-c-F_ggcatcgtggacgtctcta and BV/Runx1-mini-c-R_cggctcctaccagttctcc.

### 2.4. Rat Coccygeal Disc Degeneration Model, Hyaluronan Encapsulation, BMSCs Transplantation, and Radiographic Imaging

To confirm BV transduced BMSCs encapsulated by hyaluronan for subsequent animal transplantation, BMSCs were incubated and conjugated with hyaluronan through CD44 binding for confocal microscopic observation [29]. Briefly, the lyophilized hyaluronan (Cat. FCH-SU, Kikkoman, Japan) was used to prepare the biotinylated hyaluronan for conjugation with Alexa488. Lyophilized HA was dissolved in 50 mM boric acid, pH 5.2, at 2.0 mg/mL. This was combined with biotin hydrazide in a 20:1 weight ratio. N-(3-dimethylaminopropyl)-N’-ethylcarbodiimide hydrochloride (EDC) was added to a final concentration of 100 mM). The reaction was continued to proceed at room temperature for 16 h, after which the product was dialyzed in ddH_2_O in M.W. 12K cut-off to remove unreacted biotin hydrazide and EDC. Hyaluronan-biotin was lyophilized and stored at −20 °C for later use. The biotinylated hyaluronan was lysed in 0.1M acid and subjected to 400MHz 1H NMR (Bruker, Germany) to estimate the conjugation efficiency of biotin. To conjugate Alexa488, biotinylated hyaluronan in 10 mg/mL was incubated avidin-Alexa488 (Cat. A21370, ThermoFisher Scientific, USA) in 10 μg/mL in PBS, again dialyzed to remove unreacted Alexa488 dyes, and lyophilized, final adjusted to 1% in PBS for subsequent animal transplantation. Meanwhile, BMSCs were collected to a microtube, labeled with Dil3 fluorescent dye (Cat. D3911, ThermoFisher Scientific, USA) according to the manufacturer’s instruction and incubated with Alexa488 labeled hyaluronan solution at 37 °C for 30 min. Subsequently, the hyaluronan encapsulated BMSCs were dropped onto a slide and immediately observed by confocal microscope (Zeiss LSM 700, Berlin, Germany), 3D images were reconstructed by Image J Fiji (NIH).

For animal transplantation, the 10 to 12-week-old male SD rats (Lasco, Yi-Lan, Taiwan) were anesthetized by inhalation of 2.5% isoflurane (TERRELL™, Piramal Critical Care Inc., Bethlehem, PA, USA), and placed in a prone position on the stereotaxic instrument (Narishige Group). A 1.0 to 1.5 cm sagittal incision was made on the tail to expose the coccygeal disc co4-5, and the surgery area was treated with 0.1% Adrenalin (Cat. B060234, China Chemical and Pharmaceutical Co., Taipei, Taiwan) which largely ceased bleeding. Under a microscope, the rat coccygeal disc co4-5 was punctured transversally with 20G needle and subsequently injected with 5 × 10^5^ mock or BV transduced BMSCs encapsulated in 1% hyaluronan as previously described. A total of 6 μL of hyaluronan mixed BMSCs were injected using 30G micro-injection needle at a 1 μL/min speed through the larger track created by the previous 20G needle puncture. The facia and skin of the lesion were closed with 6-0 and 4-0 sutures, respectively. For radiographic imaging, rats were anesthetized by intramuscular injection of Zoletil 50/Rompon (1:1) in a dose of 4 mg Zoletil/kg and placed in a prone position in the radiographic instrument. X-ray images were captured at a distance of 60 cm from the tail (40 kVp, 5 mAs), and images were used for the evaluation of disc shrinkage at post-injection, represented as disc height index (DHI) [30]. 

### 2.5. Histological and Immunohistochemical Staining

Rats were sacrificed at 4- and 12-weeks post-injection, coccygeal discs co4-5 were removed, fixed with 4% paraformaldehyde (PFA) in PBS, decalcified in 0.5 M EDTA for 2 weeks, embedded in paraffin, and serially sectioned in 5 μm thickness for H&E staining. Sagittal serial sections in the mid-zone of the vertebral body were prepared for immunohistochemical (IHC) staining by standard protocol. Briefly, slides were de-paraffined, washed, blocked, and immunostained with rabbit anti-rat Runx1 (Invitrogen, US. Cat. PA5-85543), type II collagen (Santa Cruz, US., Cat. sc-28887), SOX9 (Santa Cruz. US., Cat. sc-20095), CD4 (Bioss Antibodies. Cat. bs-0647R) and CD8 (Bioss Antibodies. Cat. bs-0648R) primary antibody at 4 °C overnight, then stained with Alexa 488-conjugated goat anti-rabbit secondary antibody (Abcam. Cat. ab150077) at room temperature for 1 h, then subsequently counterstained with DAPI (VECTASHIELD^®^, VECTOR Laboratories, Inc. Cat. H-1200-10) and observed by fluorescence microscope (ImageXpress Pico, Molecular Device, USA). For histochemical staining to observe FLAG signals, the de-paraffined sections were rehydrated and incubated with antibodies to FLAG (F1804, M2; Sigma-Aldrich, St. Louis, MO, USA) and visualized by the reaction of peroxidase and diaminobenzodine (DAB). The histomorphometric analysis of H&E staining was quantified by three independent pathologists and circled the region-of-interest (ROI) by ImageJ Fiji (NIH).

### 2.6. Statistical Analysis

Data are presented as means ± SDs, statistical comparisons were performed by Student’s t-test or one-way analysis of variance (ANOVA) and *p* values < 0.05 were considered significant, represented as * *p* < 0.05; ** *p* < 0.01, *** *p* < 0.001. All calculations were performed using Statistics Analysis System (SAS) licensed to China Medical University. All in vivo data are representative of at least 3 independent experiments as indicated.

## 3. Results

### 3.1. Sustained Runx1 Expression Mediated by Cre/LoxP Recombinant Baculoviral Vector

**Figure 1:**
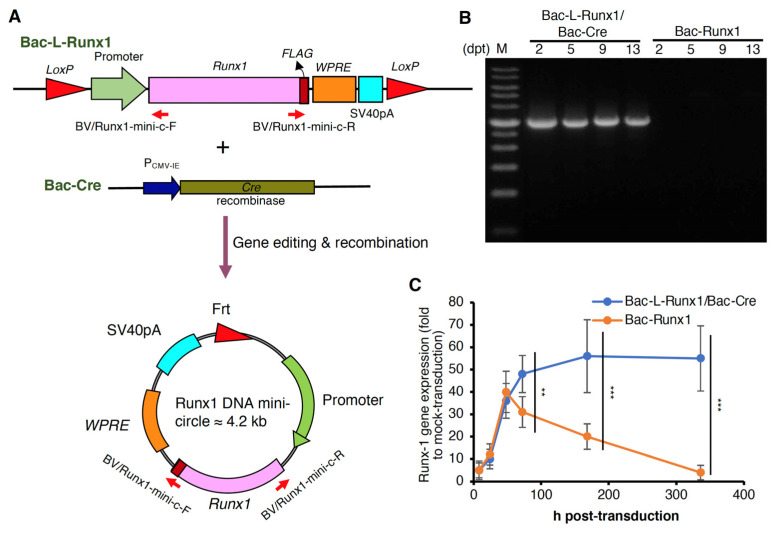
Cre/LoxP BV mediated genetic recombination, and DNA mini-circle formation efficiently sustained the transgene expression. (**A**) Illustration of Cre/LoxP BV medicated gene editing and DNA mini-circle formation. Red arrows indicate the primer pairs used to detect the DNA mini-circle formation. (**B**) The DNA mini-circle formation at 2- to 13-dpt was examined by PCR. Gene fragment was amplified through PCR reaction using BV/Runx1-mini-C-F and BV/Runx1-mini-C-R primer pairs. PCR amplicon ≈ 3085 bp. (**C**) The *runx1* gene expression of recombinant BV transduction from 8 to 336 h post-transduction was examined by qRT-PCR. (*n* = 3) ** *p* < 0.01; *** *p* < 0.001.

The MSCs isolated from the bone marrow mixture have been examined the CD markers as previously described [27]. To further confirm the functionality of BMSCs, the osteogenesis and chondrogenesis induced by culture medium alone were examined. The positive alizarin red and alcian blue staining demonstrate the differentiation capability of BMSCs isolated from our methodology (Figure S1). To examine the transduction efficiency of recombinant BV in BMSCs, we first transduced BMSCs with BV-EGFP in MOI 100, and measured the green fluorescence at days post-transduction (dpt) 2. Data reveals homogeneous GFP expression (Figure S2A) with transduction efficiency (TE) reached > 90% and mean fluorescence intensity (MFI) > 4500 (Figure S2B). We hypothesized if the bacmid was transduced to target cells, further scissored and recombined into a mini-circle, which might extend the transgene expression period. To confirm the DNA mini-circle formation and sustained transgene expression, the *Luc2* cDNA was constructed in a recombinant BV flanked by two *Lox*P recognition sites (the gene map is represented in Figure S3, the *Runx1* sequence was replaced with *Luc2* cDNA), termed as Bac-L-Luc2. Subsequently, the MSCs were transduced with Bac-L-Luc2 and Bac-Cre at MOI 100, respectively, followed by luciferase examination at 2-dpt. Data reveals the luciferase expression in Bac-Luc2 transduction reached the highest at 2-dpt and gradually decreased to near the background level at 5-dpt, similar to the control group. Conversely, luciferase expression in Bac-L-Luc2/Bac-Cre transduction reached the highest at similar 2-dpt and continued to at least 13-dpt, significantly higher than that of the Bac-Luc2 transduction (Figure S4). Identical to Luc2 cDNA construction in Cre/LoxP recombinant BV, the runx1 cDNA was constructed as illustrated (Figure S3) and termed as Bac-L-Runx1. The Cre/LoxP machinery-mediated gene targeting and recombination to form the DNA mini-circle were also schemed (Figure 1A). To confirm the DNA mini-circle formation after co-transduction of Bac-L-Runx1 and Bac-Cre, primer pairs (Figure S3) were designed for semi-quantitative PCR. The amplicon would be successfully amplified if the Bacmid proceeded recombination to form the mini-circle. Data reveals the runx1 expressing DNA mini-circles formed at 2-dpt and persisted to >13-dpt (Figure 1B). Furthermore, runx1 transgene expression in BMSCs was also detected by qRT-PCR, showing Bac-L-Runx1 transduction expressed significantly higher and more persistent runx1 mRNA than that of the Bac-Runx1 transduction (Figure 1C).

**Figure 2:**
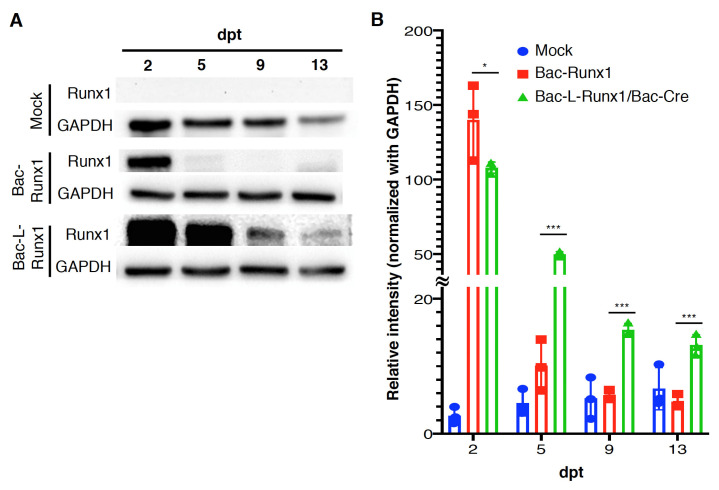
Cre/LoxP mechanism-mediated baculoviral vector recombination efficiently sustained the runx1 recombinant protein production. (**A**) Runx1 transcription factor expression at 2- to 13-dpt was examined by western blot. (**B**) Comparison of runx1 expression, protein blots intensity was normalized with GAPDH. (*n* = 3) * *p* < 0.05; *** *p* < 0.001.

The sustained expression of runx1 from Bac-L-Runx1 transduction in MSCs was measured by western blot, which persisted in expressing until 13-dpt, superior to that of the Bac-Runx1 transduction (Figure 2A). The quantitative data shows the expression levels of Bac-L-Runx1 transduction higher than that of the Bac-Runx1 transduction at least 3-fold through 5- to 13-dpt (Figure 2B). 

### 3.2. Cre/LoxP Recombinant Baculoviral Vector Elicited Low Cytotoxicity and Safety Concerns

**Figure 3:**
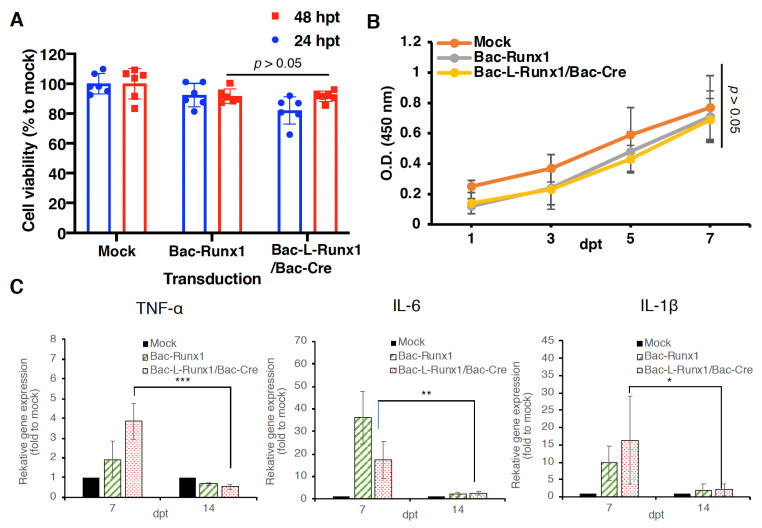
Recombinant BV was sustaining runx1 expression through Cre/LoxP mechanism possessing highly biosafety. (**A**) BMSCs’ viability at post-transduction was examined by MTT assay. (**B**) BMSCs’ proliferation rate at post-transduction was examined by WST-1 assay. (**C**) *TNF-α*, *IL-6*, and *IL-1β* mRNA expression at 7- and 14-dpt. (*n* ≥ 3) * *p* < 0.05; ** *p* < 0.01; *** *p* < 0.001.

To address the safety concern of dual recombinant BV transduction utilizing Cre/LoxP gene recognition and recombination system, we first evaluated the cell viability and proliferation rate at BV post-transduction. Compared to the control group with mock-transduction, long-termed BV transduction using Bac-L-Runx1 and Bac-Cre at MOI 100 shows very mild cytotoxicity, the cell viability remains ≈82% and 93% at 24 h and 48 h post-transduction, and shows no significant difference with short-termed BV transduction using Bac-Runx1 at MOI 100 (Figure 3A). Meanwhile, the proliferation rate of either long-termed or short-termed BV transduction shows an almost similar proliferation rate compared with the mock-transduction group (Figure 3B). Moreover, we further analyzed proinflammatory cytokine mRNA expression at BV post-transduction from 7-dpt to 14-dpt using qRT-PCR, reveals TNF-α, IL6 and IL-1β gene expression increased at 7-dpt and sharply decreased to the relative background level, almost identical to the mock-transduction group at 14-dpt at both Cre/LoxP dual BV transduction and Bac-Runx1 alone transduction (Figure 3C). Throughout the whole post-transduction period from 24 h to 14 days, BMSCs phenotypes were captured at multi areas, the representative phenotypes revealed no difference between Cre/LoxP dual BV transduction and Bac-Runx1 alone transduction, and all BMSCs in three groups showed identical phenotypes after 24 h post-transduction (Figure S5). 

### 3.3. Chondrogenesis of BMSCs Induced by runx1 Expressed Baculoviral Vector Transduction

**Figure 4:**
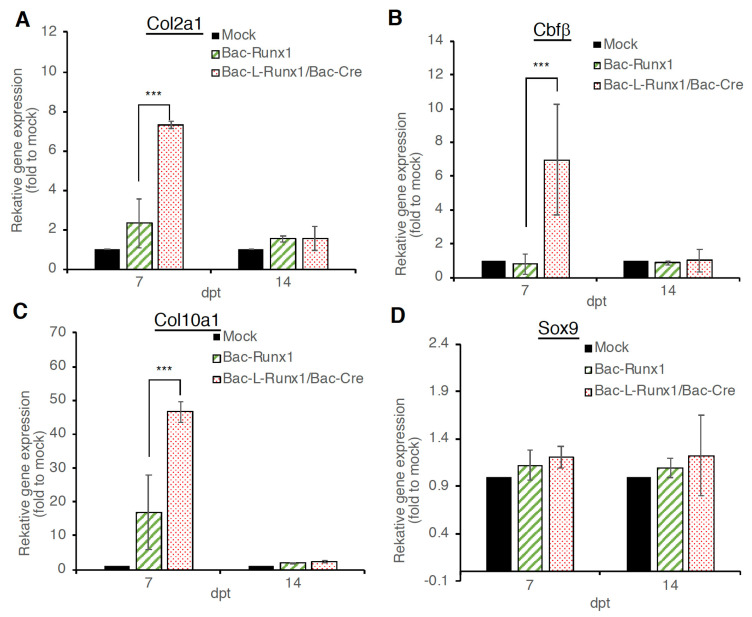
Chondrogenic markers expression at Bac-L-Runx1/Bac-Cre transduced BMSCs. (**A**) *Collagen type 2A1 (Col2a1)* mRNA expression. (**B**) *Cbfβ* mRNA expression. (**C**) *Collagen type 10A1 (Col10a1)* mRNA expression. (**D**) *Sox9* mRNA expression. BMSCs were transduced with Bac-Runx1 or Bac-L-Runx1/Bac-Cre and compared with mock-transduction. Cells were collected at 7- and 14-dpt. (*n* = 3) *** *p* < 0.001.

To examine whether the sustained runx1 transcription factor expression in BMSCs using Cre/LoxP recombinant BV transduction system inducing more efficient chon-drogenesis, mRNA was collected from BMSCs at 7- and 14-dpt and subjected to chon-drogenic markers examination. Compared to the mock-transduction group, Bac-L-Runx1 transduction triggered apparent *col2a1* and *Cbfβ* gene expression, which is significantly higher than the Bac-Runx1 transduction (Figure 4A,B) at 7-dpt, and slightly higher expression at 14-dpt but no statistical difference. However, the col10a1 also in-creased after BV transduction at 7-dpt and returned to the background level compared with the mock transduction group (Figure 4C). Whether the higher expression levels of *col10a1* attributed to more robust runx1 expression needs further detailed exploration. Meanwhile, both Bac-Runx1 and Bac-L-Runx1/Bac-Cre transductions elicited higher *sox9* expression than mock transduction, but no statistical difference between Bac-Runx1 and Bac-L-Runx1/Bac-Cre transductions (Figure 4D). Notably, the elevation of *col2a1* and *Cbfβ* gene expression, demonstrated sustained runx1 transcription factor expression directly promotes chondrogenesis of BMSCs.

### 3.4. Disc Height Shrinkage Ameliorated by runx1 Overexpressed BMSCs Transplantation in a Disc Degeneration Model in the Rat

**Figure 5:**
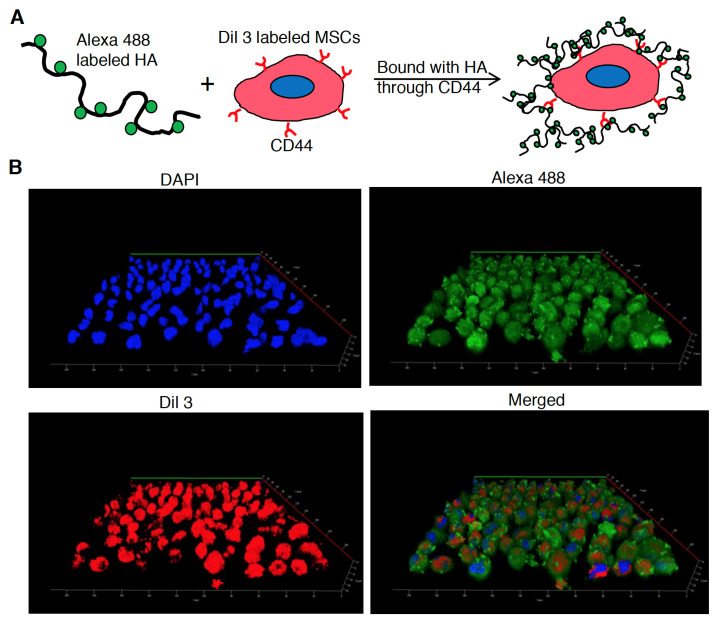
BV transduced BMSCs were encapsulated by hyaluronan for grafting in a punctured disc. (**A**) Scheme of Bac-Runx1, Bac-L-Runx1/Bac-Cre or mock transduced BMSCs bound with hyaluronan through CD44 receptor. For confocal microscope observation, hyaluronan was labeled with Alexa488, and BMSCs were marked with Dil3, respectively. BMSCs and hyaluronan were incubated at 37 °C, 30 min, followed by microscopic observation. (**B**) Representative images show the Bac-L-Runx1/Bac-Cre transduced BMSCs conjugated with hyaluronan and immediately observed in suspension status.

To supply a jelly-like material and anabolic factor engineered BMSCs into a damaged disc to compensate for the ECM and NP loss in a progressive disc degenerative scenario, the hyaluronan was considered as a carrier for BMSCs grafting through binding with CD44 on BMSCs as previously described [29]. Meanwhile, hyaluronan was one of the essential components and ECM composition in a healthy disc [31]. Furthermore, the bounding capability should be addressed to examine whether the hyaluronan can be served as a carrier to trap and deliver cells into IVD. Therefore, BMSCs were transduced with Bac-Runx1 or Bac-L-Runx1/Bac-Cre and compared with mock transduction. Prior BMSCs grafting in rat disc, the hyaluronan encapsulated BMSCs were examined by confocal microscopy. Hyaluronan was labeled with Alexa488. Meanwhile, BMSCs were labeled with Dil3 to emit the cytosol, followed by incubation with hyaluronan for cell encapsulation (Figure 5A). Immediately, the BMSCs were observed in suspension status. The red fluorescence represents the cells in a spherical morphology, and the green fluorescence covers the spherical cells represent the cells sophisticatedly encapsulated by the hyaluronan (Figure 5B), which is the most abundant component in the ECM of IVD.

**Figure 6:**
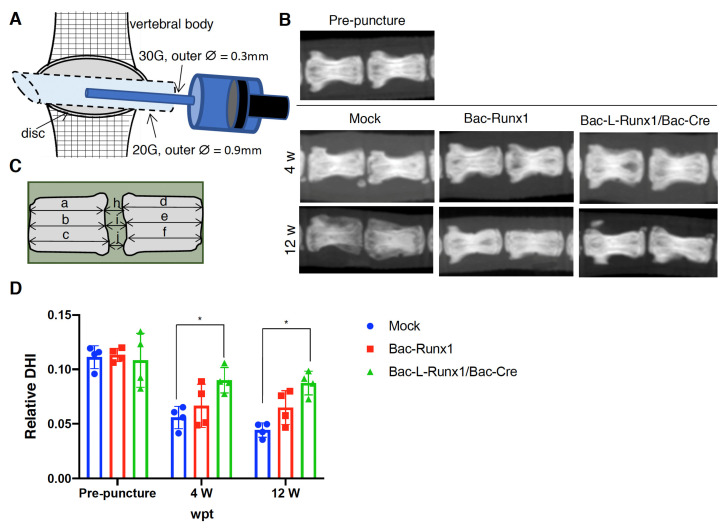
Radiographic images evaluate the disc height index (DHI) after grafting the recombinant BV transduced BMSCs in the punctured coccygeal disc in the rat. (**A**) Illustration of disc puncture and cells delivery method. (**B**) X-ray radiographic images showed the coccygeal disc height at BMSCs post-injection 4 w and 12 w compared with pre-puncture. (**C**) DHI measurement is illustrated. DHI = 2 × (h + i + j)/(a + b + c + d + e + f). (**D**) Change of relative DHI at recombinant BV transduced BMSCs post-injection. Relative DHI was represented as post-puncture DHI/pre-puncture DHI. (*n* ≥ 3) * *p* < 0.05; *p* < 0.001, ∅ is diameter.

Since the sustained runx1 expression in BMSCs promotes the Collagen II and ECM-related components to preserve the elastic property and maintain the hydration content possessed by a healthy spinal disc to provide the disc space. Therefore, to examine the sustained expression of runx1 to ameliorate the disc degeneration scenario, a punctured disc in a rat model was transplanted with Bac-L-Runx1 or Bac-Runx1 transduced BMSCs, followed by radiographic imaging. To create damage in the disc, we punctured the coccygeal disc with 20G needle and immediately micro-injected BV genetically modified BMSCs with 30G needle in a very slow flow rate through the identical path as illustrated (Figure 6A), which ensured whole BMSCs were reserved in the punctured cavity. X-ray images were captured at 4- and 12-weeks post-transplantation (wpt), showing a slightly shorter disc height in the Mock and Bac-Runx1 group than that of the Bac-L-Runx1 group (Figure 6B). Therefore, the disc height difference from X-ray images was sophisticatedly measured as illustrated (Figure 6C) and represented as disc height index (DHI) for comparison, based on the following equation DHI = 2 × (h + i + j)/(a + b + c + d + e + f). Data reveals that only Bac-L-Runx1 transduction ameliorated disc height shrinkage compared to mock-transduction showed a significant difference at 4 wpt and 12 wpt (Figure 6D).

### 3.5. Cre/LoxP Recombinant Baculoviral Vector-Mediated runx1 Sustained Expression Ameliorated Disc Degeneration Progress and ECM Loss

**Figure 7:**
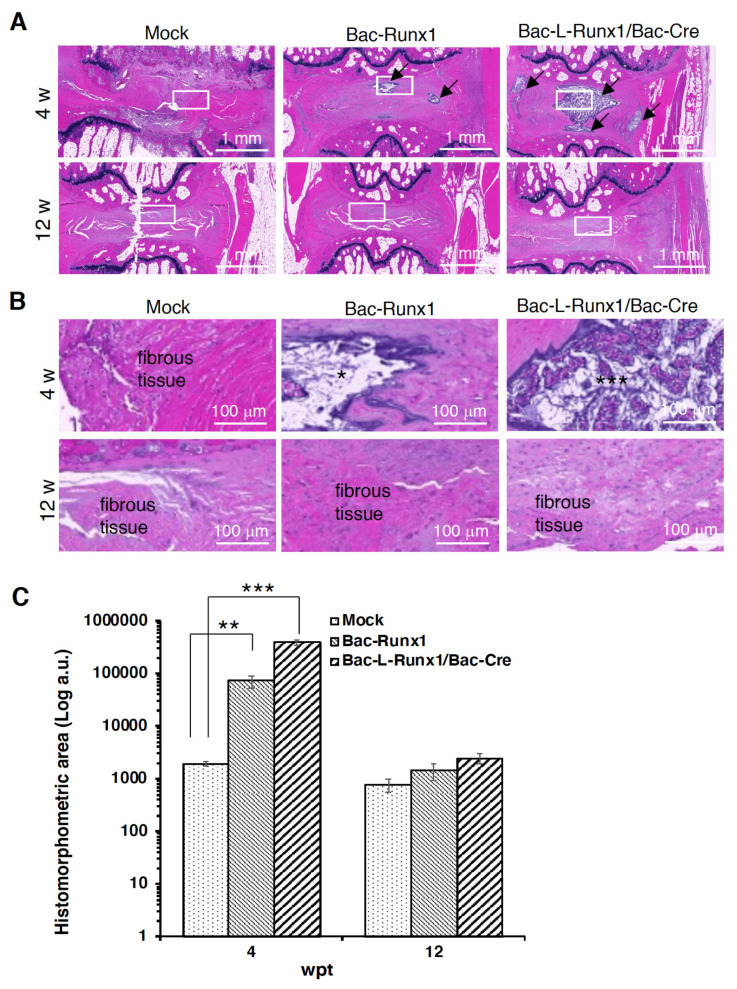
Histological examination of disc degeneration transplanted with BV transduced BMSCs and histomorphometric analysis. (**A**) Representative H&E staining at low magnification. Arrows indicate the gelatinous materials in the disc core. (**B**) H&E staining showed the area magnified from the solid line box at (**A**). Stars indicate the jelly-like materials secreted from NPs. (**C**) Histomorphometric analysis of gelatinous materials in H&E staining from 3 groups. Five sections in the middle part of serial disc sections from each group were picked up for analysis. (Weeks post-transplantation, wpt) (*n* = 3) ** *p* < 0.01; *** *p* < 0.001.

To examine the ECM content in the punctured disc with BV-modified BMSCs transplantation, the H&E and immunohistological staining were conducted at 4- and 12-wpt. Data shows apparent fibrous tissue infiltration in the Bac-Runx1 and mock transduction groups at either 4- or 12-wpt (Figure 7A,B) compared to an intact disc (Figure S6). Conversely, the Bac-L-Runx1 transduction shows jelly-like transparent ECM that remained inside the laminar-shaped disc ring at 4-wpt and gradually lost it to remain a very sparse ECM at 12-wpt (Figure 7A,B). Histomorphometric analysis reveals more abundant ECM in the Bac-L-Runx1/Bac-Cre transduction group compared with the mock-transduction group (*p* < 0.001) at 4-wpt (Figure 7C). Although the Bac-L-Runx1 transduction group did not ultimately rescue the punctured disc and recover it to near intact disc, the hydration content and ECM were higher reserved in the Bac-L-Runx1 group than that of the Bac-Runx1 or mock group (Figure 7B,C). Furthermore, to distinguish the ECM regeneration attributed from the endogenous runx1 stimulation or recombinant BV mediated exogenous runx1 overexpression, the FLAG tag fused to runx1 was examined (see the gene map in Figure 1).

**Figure 8:**
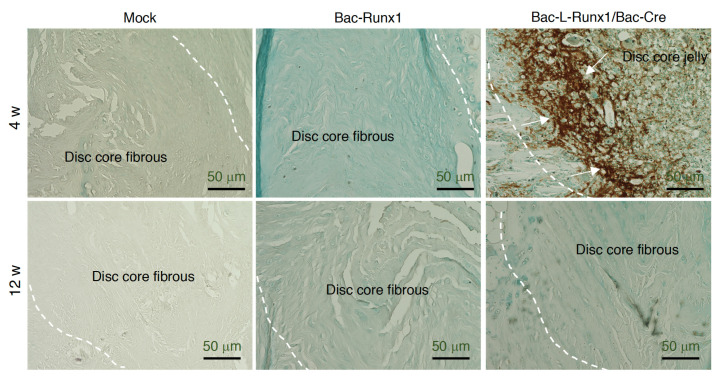
Immunohistological staining to detect the FLAG signals. Recombinant runx1 expressed from Bac-Runx1 or Bac-L-Runx1/Bac-Cre transduction was detected by FLAG signals to discriminate the exogeneous runx1 from BMSCs’ endogenous runx1 expression. At 4 w and 12 w post-transplantation, sections retrieved from the middle part of the disc were immunostained with 1st antibody against FLAG, followed by HRP and DAB staining. A dashed line indicates the tissue margin between AF and NP. Arrows indicate the deep intensity of DAB staining in the disc core.

The serial slides from the mid-section retrieved from the damaged discs were subjected to immunohistochemical staining using 1st antibody against the FLAG. Data shows almost no FLAG signal in the Mock and Bac-Runx1 groups at 4- and 12-wpt (Figure 8). The jelly-like materials in the disc core of the Bac-L-Runx1 group show abundant FLAG staining signals at 4-wpt but extinguish to low leveled signals at 12-wpt (Figure 8). Data demonstrates the overexpressed exogenous runx1 derived from BV transduced BMSCs allotransplantation. 

**Figure 9:**
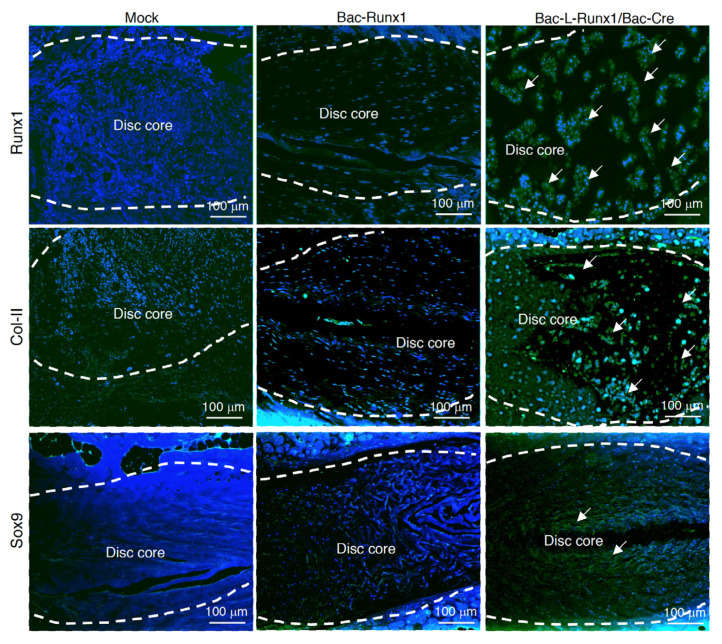
Jelly-like materials loss in the disc core was examined by immunohistochemical (IHC) staining. Discs were collected at 4 weeks post-transplantation, and the representative data shows the IHC staining using runx1, collagen type II, and sox9 1st antibodies, followed by Alexa488 conjugated 2nd antibody and DAPI staining. Runx1, collagen type II, and sox9 protein were maintained in the disc core at Bac-L-Runx1/Bac-Cre transduction, compared with Bac-Runx1 and mock transduction. A dashed line indicates the tissue margin between AF and NP, circles the disc core matrix. Arrows indicate the positive signals stained in the disc core. (*n* = 3).

Moreover, the immunohistochemical staining shows runx1 recombinant protein was expressed in the punctured disc space, attributed from Bac-L-Runx1 and BMSCs transplantation at 4-wpt (Figure 9). Furthermore, the collagen II and sox9 proteins, both critical components of a healthy disc, were stained in higher color intensity in the Bac-L-Runx1 group compared to that of the Bac-Runx1 or mock group at 4-wpt (Figure 9). Again, it demonstrated that the Bac-L-Runx1 merged with BMSCs engineering holds the superior capability to regenerate damaged discs.

### 3.6. Transplantation of Cre/LoxP Recombinant Baculoviral Vector Modified BMSCs Elicited Sparse Adaptive Immune Responses

**Figure 10:**
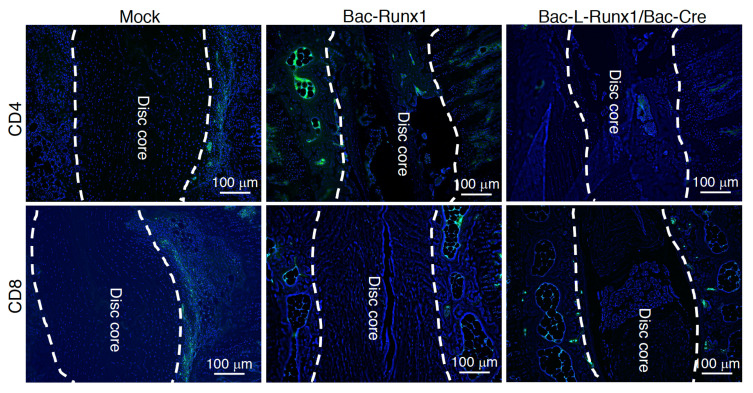
Adaptive immunity was evaluated by infiltration of CD4^+^ and CD8^+^ T cells. Traumatic disc transplanted with BMSCs genetically modified by Bac-L-Runx1/Bac-Cre were compared with Bac-Runx1 or mock-transduction. Representative images show the IHC staining using anti-CD4 and anti-CD8 1st antibodies, followed by 2nd antibody labeled with Alexa488, count-stained with DAPI. All groups were collected at 4 weeks post-transplantation. Dashed line circles the disc core matrix. (*n* = 3).

The runx1 overexpressed BMSCs transplantation showed a tremendous difference in the jelly-like materials in disc core at 4-wpt rather than 12-wpt. Therefore, to examine the immune responses elicited from BV modified allogeneic BMSCs transplantation, the slides of animals at 4-wpt were immunohistochemically stained with anti-CD4 and anti-CD8 1st antibodies to detect the CD4^+^ and CD8^+^ T-cells, respectively, which represent the adaptive immunities [32,33]. Compared with mock-transduction, data shows very few CD4 and CD8 positive signals were detected in either Bac-Runx1 or Bac-L-Runx1/Bac-Cre transduction groups (Figure 10), representing sparse cellular immunity and memorial T-cells infiltration were elicited at post-transplantation. Again, it demonstrated the minimum safety concerns of BV mediated Cre/LoxP gene-editing for runx1 overexpression. But, the long-termed safety and immune characteristics should be addressed through more detailed and thorough studies. 

## 4. Discussion

With global health technology advancement and human life extension, spine-related disorders emerged as one of the leading causes of disability and financial burden worldwide. IVD herniation is one of the most prevalent causes and spine disorders, leading to motion disability and inexplicable back pain and causing patients to seek many complicated non-operative and operative treatments. Furthermore, the patient’s other morbidity, which may accelerate the disc degeneration to worse. Nevertheless, some surgeries addressed disc herniation and corruption but conflicted with conservative treatments, which would not completely replace the disc. Total disc arthroplasty can preserve the motion segment; however, the long-term results of this surgical treatment are still in question. Such as, degeneration of adjacent segments was occurred frequently after fusion surgeries, regardless of patient age [34,35,36,37]. Furthermore, the removal of NP tissue during discectomy accelerates degeneration [38]. These tremendous unmet medical needs drive many emerging technologies development, such as artificial disc replacement using biomolecular drugs loaded and 3D-printed synthetic cages, providing promising therapeutic outcomes. Furthermore, the non-invasive biological approaches are focused on cell-based or gene therapies, gain more and more attention and promote many preclinical or clinical settings.

The IVD is one of the avascular tissues in the human body, such as the hyaline cartilage in joint, yet it is a highly hydrated tissue because of the composition and structure of its ECM. The ECM surrounding NP cells are composed of type II collagen (Col2a1), GAGs, and proteoglycans such as aggrecan and is similar to that of the composition in hyaline cartilage [10,39]. The IVD is a dynamic tissue, the ECM undergoes continuous remodeling as IVD cells produce new ECM material and proteases degrade old material [40]. Disc degeneration is not only an aging disorder but is also related to the shortage of critical factors involved in the homeostasis of the disc matrix [41]. Overall, disc degeneration is defined as the disruption of the equilibrium between the anabolism and catabolism of the disc matrix [39]. Similarly, disrupted homeostasis exists in chondrocytes in osteoarthritis, and current therapeutic strategies aim to alter this misbalanced homeostasis [42]. Therefore, we hypothesize that similar therapeutic techniques and anabolic factors can be applied to treat disc degeneration, mainly induced by traumatic injury, which needs the excellent and real-time supplement of disc ECM to support the disc structure.

Runx1 has been shown to play a critical role in MSC chondrogenesis, is involved in the proliferation and survival of chondrocytes [11,12], and plays a direct role in kartogenin mediated cartilage repair [9]. Runx1 overexpression has been demonstrated that potently enhanced col2a1 expression and GAG content in OA joint, and evidenced that runx1 binds with Col2A1 promoter responsive elements [10]. To increase the ECM secretion in the damaged disc to compensate for the trauma-induced ECM loss and preserve the damaged disc through a tissue-engineered approach, we combined the runx1 expression with BMSCs delivery. Through Cre/LoxP BV mediated gene recombination possessing high transduction efficiency (Figure S2), the runx1 was persistently expressed in BMSCs, followed by sophisticatedly encapsulated in hyaluronan for transplantation in a traumatic disc (Figure 5), and examined the healing efficiency with radiographic imaging and histological analysis. In the current study, two recombinant BVs used for Luc2 and runx1 delivery were constructed. The Cre/LoxP mediated transgene editing scenario exhibited superior reporter and transcription factor expression efficiency in the Cre/LoxP BV modified BMSCs than the BMSCs modified by conventional BV (Figure 2 and Figure S4). Runx1 expression was detected for at least 13-dpt, which may be due to the higher stability and stealth property provided by the DNA mini-circles derived from Cre/LoxP conducted transgene recombination (Figure 1). Furthermore, we have demonstrated that Cre/LoxP BV mediated persistent runx1 overexpression could be used to mitigate disc shrinkage (Figure 6) and ECM loss (Figure 7 and Figure 9). The in vitro transduction data demonstrates the particular ECM elements boosted from persistent runx1 overexpression (Figure 4). Remarkably, the *col10a1* was also enhanced, which may be due to the runx1 overexpression leading to a significant and progressive upregulation of both runx2 isoforms. Concomitant with the highest induction of *runx2* transcripts, leading to enhancement of the *type X collagen* mRNA levels. These results reveal that runx1 plays a role in mesenchymal stem cell commitment to the cartilaginous lineage, showing the intricacy of runx-mediated regulation of chondrogenesis [11]. 

One preliminary design is the traumatic disc damage model, which is distinct from the chronic degeneration disease (DDD) in humans and promotes us to survey the physical loading or drug-induced disc damage model to examine our concept in the future study [43,44]. Despite differences in rat and human disc degeneration, it is clear that the needle puncture injury leads to disc degeneration and shrinkage in the absence of medical treatment, and sustained runx1 supplementation slowed the rate of disc shrinkage (Figure 6). Meanwhile, the sustained runx1 expression attributed to Cre/LoxP BV mediated DNA mini-circle recombination and persistent exogeneous runx1 overexpression in the disc (Figure 7). Therefore, in future work, we are encouraged to combine the fibrous cartilage homing peptides, such as many targeting peptides utilized in the drug delivery [45,46,47], and hyaluronan to manufacture an IVD specific targeting cell carrier for the genetically modified MSCs delivery, aimed to regenerate the damaged disc completely. 

Transplantation of Cre/LoxP mechanism gene-edited BMSCs by BV transduction for spinal disc healing holds several advantages: (1) BMSCs were in vitro transduced by recombinant BV, and transplanted to the animal at 1-dpt at least, vastly decreased the possibility of non-targeted cells transduced by BV when directly administration in vivo and ameliorated the safety uncertainty [32,48,49,50]. (2) The cytosolic recombinant protein such as runx1, a transcription factor expressed explicitly in cytosol rather than secretory protein, inevitably should be expressed via cells genetically modification such as BV transduction. This also restricts the recombinant protein expressed in the cytosol rather than circulates physiologically. (3) The sustained runx1 expression through Cre/LoxP gene-editing mediated by BV holds highly genetic recognition, decreases the off-target gene editing possibility (Figure 1). (4) The lower proinflammatory cytokines expression (Figure 3) and very sparse immune cells infiltration (Figure 9) at BV post-transduction demonstrated the safety of Cre/LoxP BV used in prolonging the transgene expression. Furthermore, T cell-mediated cellular immune responses are the primary cause of failure of MSCs allotransplantation for regenerative medicine [51]. The processing of donor cells’ allogeneic MHC molecules by host antigen-presenting cells (APCs) may lead to allorecognition by CD4 positive T cells, while MHC class I-mediated presentation of transgene products activates the host CD8 positive T cells [33]. Compared with mock-transduction, data shows very few CD4 and CD8 positive signals were detected in either Bac-Runx1 or Bac-L-Runx1/Bac-Cre transduction groups (Figure 9), demonstrated sparse cellular immunity and memorial T-cells infiltration were elicited from Cre/LoxP BV transduction.

Overview of the current biological treatment strategies for spinal disc degeneration, including hydrogel-based grafting, multipotent cells transplantation, anabolic factor delivery, and gene and cell combined therapy, effectively mitigated the disc degeneration progress. Biological interventions aim to slow down the rate of degeneration and senescence, induction of regeneration, enhancement of viability and matrix reproduction from the residual surviving cells, and eventually restoration of mechanical properties of IVD. For further clinical application, the safety hesitates raised from biological interventions, long-termed evidence for satisfied and sufficient effectiveness, and durability of the regenerated disc should be more thoroughly addressed. 

## 5. Conclusions

The recombinant BV possessed the Cre/LoxP gene editing function, formed runx1 expressing DNA mini-circles, persisted to at least 13-dpt. Furthermore, this Cre/LoxP BV induced a more sustained runx1 gene expression and robust runx1 recombinant protein expression than the conventional BV at least 3-fold through 5- to 13-dpt, which resulted in more ECM secretion and BMSCs’ chondrogenesis. Meanwhile, the safety concern of dual recombinant BV transduction utilizing Cre/LoxP gene recognition and recombination system was also evaluated, revealed very mild pro-inflammatory cytokine expression and cytotoxicity, almost identical to the mock-transduction group at 14-dpt. Furthermore, the runx1 expressing DNA mini-circles edited by Cre/LoxP gene recognition and editing mechanism exactly sustained the runx1 expression in BMSCs, followed by encapsulation in hyaluronan and disc grafting, ameliorated the disc space shrinkage resulting from disc trauma, compared to mock-transduction showed a significant difference at 4-wpt and 12-wpt (*p* < 0.05). Moreover, the histomorphometric analysis examined the jelly-like IVD materials loss reveals more abundant ECM in the Cre/LoxP BV transduction group than the mock-transduction group (*p* < 0.001) at 4-wpt. Finally, the immunohistological examination demonstrated the robust FLAG-runx1 fusion protein expression, type-II collagen, sox9 expression, and quiescent CD4+ and CD8+ T cells infiltration in the Cre/LoxP BV transduction group, demonstrated the convinced genetic manipulation feasibility and safety. This proof-of-concept study could serve as a stepping stone to shed light on further clinical applications of MSCs’ allotransplantation using tissue targeting hyaluronan and Cre/LoxP genetic editing.

## Data Availability

The data presented in this study are available on request from the corresponding author.

## Supplementary Materials

The following supporting information can be downloaded at: www.mdpi.com/article/10.3390/biomedicines10030555/s1. Figure S1: Differentiation of BMSCs isolated from rat bone marrows; Figure S2: BV-EGFP transduction effi-ciency in rat BMSCs; Figure S3: Primers map to detect the runx1 DNA mini-circle after Bac-L-Runx1/Bac-Cre co-transduction in BMSCs; Figure S4: Luciferase expression of BMSCs transduced with Bac-Luc2 and Bac-L-Luc2/Bac-Cre, compared with mock transduction; Figure S5: BMSCs mor-phology after recombinant BV transduction; Figure S6: Intact coccygeal disc H&E staining.
