# Supplementary material for: Cre/LoxP Genetic Recombination Sustains Cartilage Anabolic Factor Expression in Hyaluronan Encapsulated MSCs Alleviates Intervertebral Disc Degeneration
Source: Biomedicines. 2022 Feb 26;10(3):555. doi: 10.3390/biomedicines10030555 (PMC8945655; doi:10.3390/biomedicines10030555)
Supplement: Supplementary file 1 [file biomedicines-10-00555-s001.zip › biomedicines-1571375-supplementary.pdf]

biomedicines

MDPI

Supplementary data

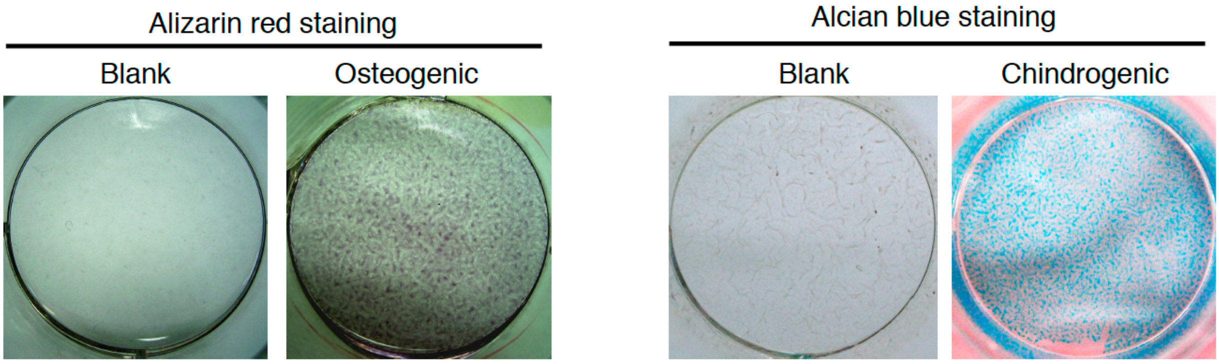

Figure S1. Differentiation of BMSCs isolated from rat bone marrows. To confirm the functionality of BMSCs induced by the osteogenic and chondrogenic medium, compared with blank BMSCs cultured in standard medium without induction. Representative images show the BMSCs stained with Alizarin Red and Alcian blue at 14 days, respectively. (N≥ 3)

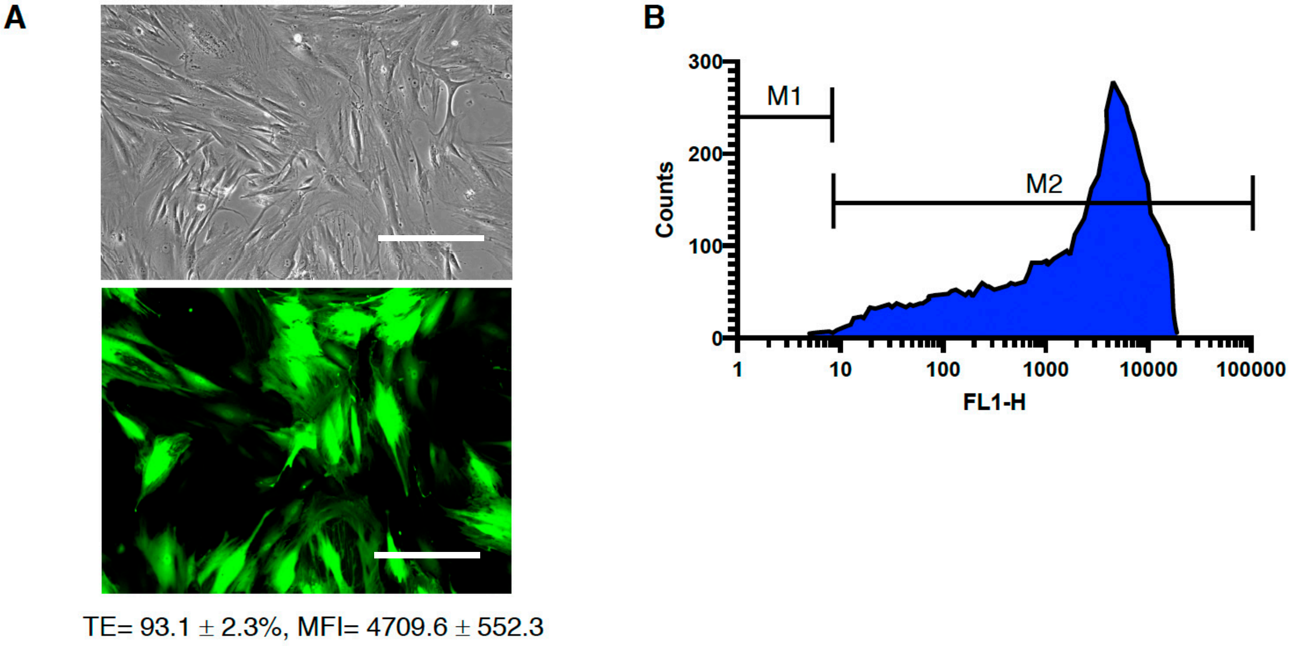

Figure S2. BV-EGFP transduction efficiency in rat BMSCs. BMSCs were transduced with BV-EGFP in MOI 100 and measured the green fluorescence at days post-transduction (dpt) 2. **(A)** Bright-field microscopic observation (up), microscopic fluorescence observation (down). **(B)** Flow cytometry analyzes BV transduction efficiency and means fluorescence. Transduction Efficiency≈ 93.1 ± 2.3%, Mean Fluorescent Intensity≈ 4709.6 ± 552.3. (N= 3) Scale bar= 100 µm.

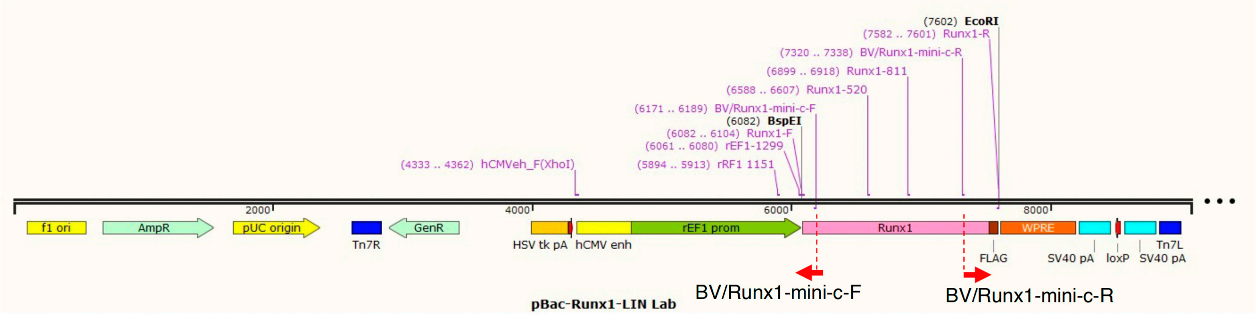

Figure S3. Primers map to detect the runx1 DNA mini-circle after Bac-L-Runx1 / Bac-Cre co-transduction in BMSCs. (A) The donor vector used to generate runx1 expressing bacmid through Bac-to-Bac® system, the runx1 coding sequence was flanked by two LoxP recognition sites. The Cre/LoxP mechanism was used to medicate the gene editing and DNA mini-circle formation. The BV/Runx1-mini-c-F and BV/Runx1-mini-c-R primer pairs were used to detect the runx1 DNA mini-circle. Primer pairs were shown in solid red arrows.

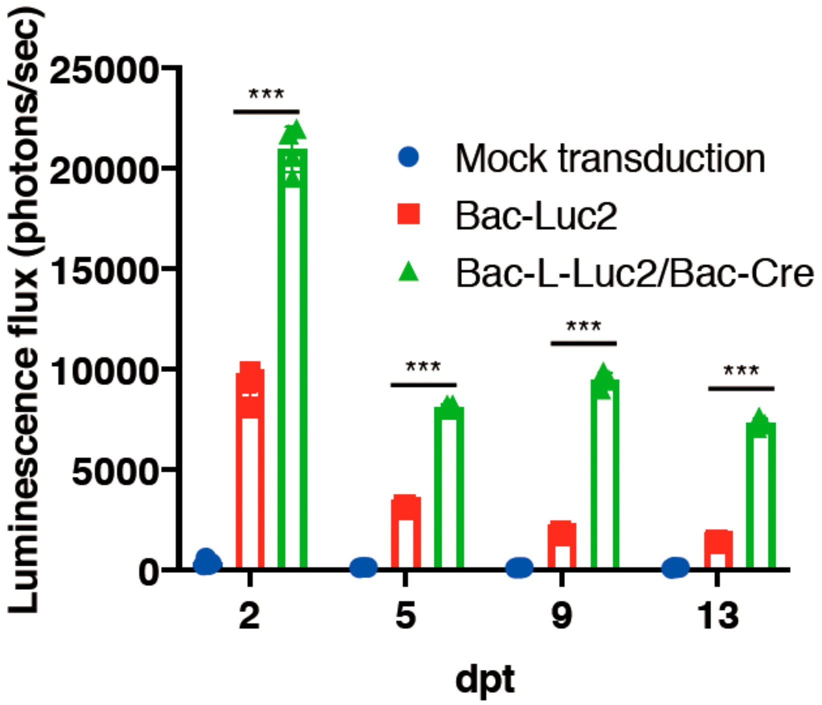

Figure S4. Luciferase expression of BMSCs transduced with Bac-Luc2 and Bac-L-Luc2/Bac-Cre, compared with mock transduction. To examine the sustained transgene expression, the *Luc2* cDNA was constructed in a recombinant BV flanked by two *Lox*P recognition sites, termed as Bac-L-Luc2. Subsequently, the BMSCs were transduced with Bac-Luc2 or Bac-L-Luc2/Bac-Cre at MOI 100, respectively, followed by luminescence examination at 2- to 13-dpt. (N≥ 3) *p<0.05; **p<0.01; ***p<0.001

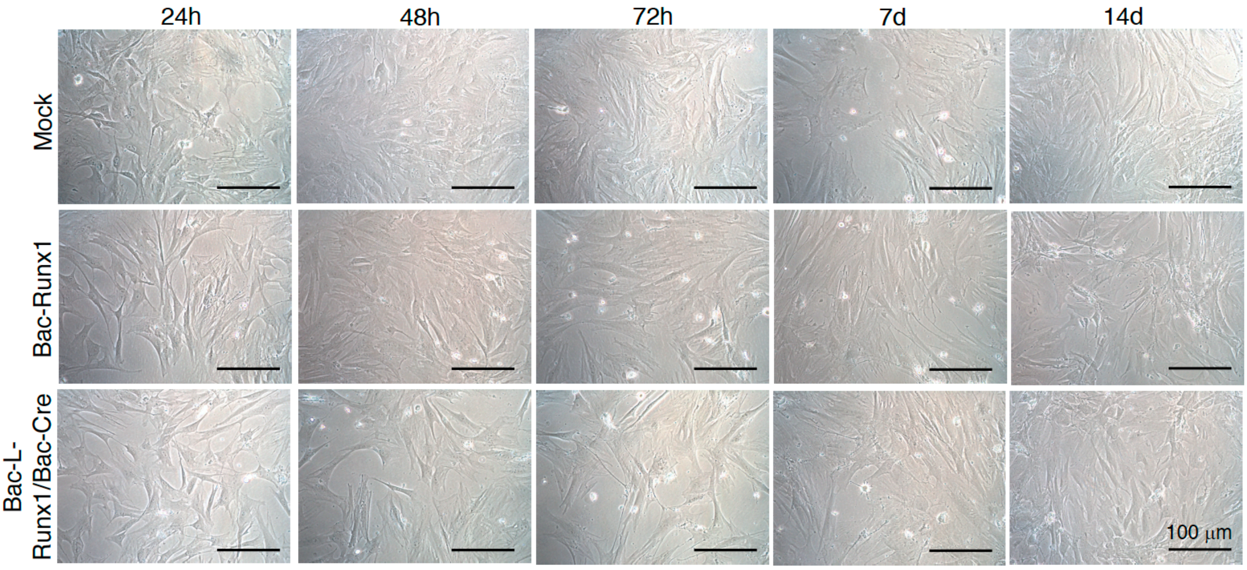

Figure S5. BMSCs morphology after recombinant BV transduction. Representative images show the morphology change of BMSCs at 24 h to 14 days post-transduction. Scale bar= 100 μm. (N≥ 3)

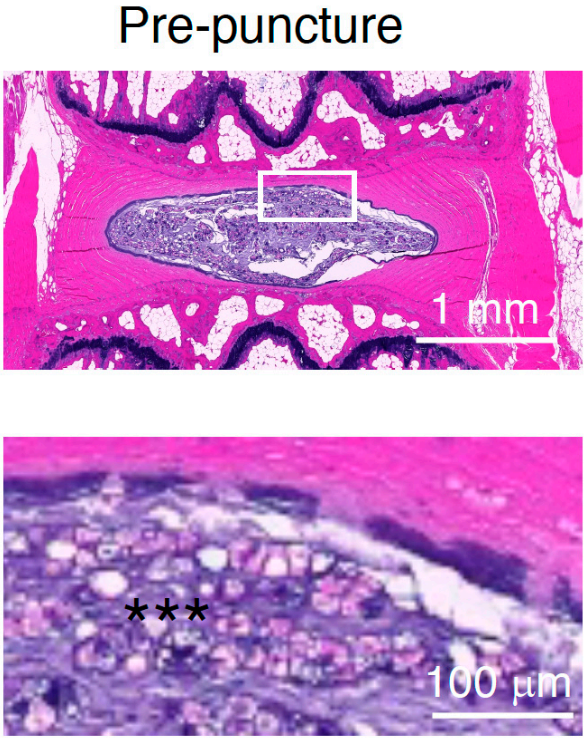

Figure S6. Intact coccygeal disc H&E staining. Low magnification shows intact AF and NP (up). The area magnified from the solid line box was placed in the bottom, stars indicate the jelly-like materials secreted from NP (bottom).
